# Comparative physiological and transcriptomics analysis revealed crucial mechanisms of silicon-mediated tolerance to iron deficiency in tomato

**DOI:** 10.3389/fpls.2022.1094451

**Published:** 2022-12-21

**Authors:** Yu Shi, Shuxun Guo, Xin Zhao, Mengzhu Xu, Jin Xu, Guoming Xing, Yi Zhang, Golam Jalal Ahammed

**Affiliations:** ^1^ College of Horticulture, Shanxi Agricultural University, Taigu, Shanxi, China; ^2^ College of Horticulture and Plant Protection, Henan University of Science and Technology, Luoyang, Henan, China; ^3^ Henan International Joint Laboratory of Stress Resistance Regulation and Safe Production of Protected Vegetables, Luoyang, Henan, China

**Keywords:** silicon, low iron, transcriptomics analysis, organic acid, sugar metabolism, oxidative stress

## Abstract

Iron (Fe) deficiency is a common abiotic stress in plants grown in alkaline soil that causes leaf chlorosis and affects root development due to low plant-available Fe concentration. Silicon (Si) is a beneficial element for plant growth and can also improve plant tolerance to abiotic stress. However, the effect of Si and regulatory mechanisms on tomato plant growth under Fe deficiency remain largely unclear. Here, we examined the effect of Si application on the photosynthetic capacity, antioxidant defense, sugar metabolism, and organic acid contents under Fe deficiency in tomato plants. The results showed that Si application promoted plant growth by increasing photosynthetic capacity, strengthening antioxidant defense, and reprogramming sugar metabolism. Transcriptomics analysis (RNA-seq) showed that Si application under Fe deficiency up-regulated the expression of genes related to antioxidant defense, carbohydrate metabolism and organic acid synthesis. In addition, Si application under Fe deficiency increased Fe distribution to leaves and roots. Combined with physiological assessment and molecular analysis, these findings suggest that Si application can effectively increase plant tolerance to low Fe stress and thus can be implicated in agronomic management of Fe deficiency for sustainable crop production. Moreover, these findings provide important information for further exploring the genes and underlying regulatory mechanisms of Si-mediated low Fe stress tolerance in crop plants.

## Introduction

Iron (Fe) is not only an essential mineral element for plants but also an activator of many vital enzymes (Chao and Chao, 2022). It is a cofactor for numerous proteases in the redox process including photosynthesis, nitrogen fixation and nucleic acid synthesis (Nikolic et al., 2019). Although Fe is abundantly present in the soil as an element, it commonly occurs in the oxidized form, which is not a suitable form for plant uptake (Ahammed et al., 2020). Notably, Fe solubility is very low in alkaline soils which causes Fe deficiency-induced chlorosis. Fe deficiency inhibits photosynthetic capacity and subsequently sucrose metabolism in plants (Nikolic et al., 2019). Moreover, Fe deficiency-induced disruption in the photosynthetic and respiratory electron transport leads to the massive production of reactive oxygen species (ROS) in chloroplasts and mitochondria (M'Sehli et al., 2014; Zaid et al., 2020).

Silicon (Si) is the second most abundant element in the earth’s crust (Khan et al., 2022). Although Si is not a well-established essential element for plant growth and development, recent studies have shown that Si is beneficial to plant growth (Gou et al., 2020; Rastogi et al., 2021). Plant roots mainly absorb silicic acid (H_4_SiO_4_), which is then transported through the xylem to shoot, eventually forming amorphous Si (SiO_2_·nH_2_O) deposited in the cell wall or intercellular space (Zhang et al., 2018; Khan et al., 2021). Dissolved Si (H_4_SiO_4_), can be readily taken up by plants and it plays an important role as an alleviator of both biotic and abiotic stress (Ahmad et al., 2019; Ahanger et al., 2020; Ahammed and Yang, 2021; Hussain et al., 2021). The absorption of Si by plants consists of active absorption and passive absorption, and both types may exist simultaneously (Ahmed et al., 2014; Zaid et al., 2018). Silicon deposition on the cell wall aids in maintaining the cellular ion balance, osmotic regulation and ROS homeostasis, thus enhancing the resistance of plants to stress (Bakhat et al., 2018).

Recent studies have shown that Si can enhance the re-transfer of Fe from old leaves to new leaves, and promote the transfer of Fe by increasing the content of Fe^2+^ and niacinamide, the chelating agent of Fe^3+^ in the phloem (Pavlovic et al., 2016). Silicon application significantly increased the total Fe content under Fe deficiency, alleviated the symptoms of leaf chlorosis caused by Fe deficiency, and promoted the growth of shoots and roots in cucumber (Bityutskii et al., 2018). Under Fe deficiency, plants treated with Si can absorb Fe faster than those treated without Si, especially Si increases the transport rate of Fe from root to stem, suggesting that the increased expression of transporters affects the absorption and transport of Fe, which is of the great significance of maintaining normal growth of plants under Fe deficiency (Peris-Felipo et al., 2020).

Despite several reports showing that Si can aid plants in Fe uptake, the ameliorative effect of Si on Fe deficiency and its physiological and molecular mechanisms still remain unclear. In this study, the tomato (*Solanum lycopersicum* L.) variety ‘Micro-Tom’ was used as experimental material in a hydroponic system to study the effects of Si root application on growth characteristics, photosynthetic fluorescence parameters, glucose metabolism, organic acid content and transcriptomics of tomato seedlings under Fe deficiency. Transcriptome analysis and physiological results explained how Si can improve the tolerance of tomato plants to Fe deficiency, which provides a theoretical basis and reference for improving the tolerance of tomato plants to Fe deficiency.

## Materials and methods

### Plant materials and treatments

Tomato (*S. lycopersicum* L. cultivar “Micro-tom”) seeds were purchased from Ball Horticultural Company in the United States. The Si source was K_2_SiO_3_·nH_2_O (containing Si 47%-51%), and the Fe source was EDTA-FeNa (containing Fe ≥13%). The nutrient solution used in hydroponics was adopted from the Japanese Yamazaki tomato formula (Qu et al., 2020; Zhang et al., 2019a). The experiment was carried out in the Artificial Climate Room, College of Horticulture, Shanxi Agricultural University, Taigu, China with various environmental parameters as follows: temperature 28/18°C (day/night), light intensity 800 μmol·m^-2^·s^-1^, and photoperiod 16/8h. Seedlings at the four-leaf stage were transplanted to hydroponic pots (28cm×19cm×14cm) containing 1/2 strength of the Japanese Yamazaki formula nutrient solution with 100 μM Fe concentration. After a week of recovery of the seedlings, the nutrient solution was replaced with full-strength Japanese Yamazaki formula consistent with different treatments. Treatments were then imposed in different combinations of Fe and Si application which resulted in the following 6 treatments: (i) Control (CK): 100 μmol/L Fe+ 0 mmol/L Si, (ii) Control and Si application (CK+Si): 100 μmol/L Fe + 1.5 mmol/L Si, (iii) Fe deficiency treatment (10 Fe): 10 μmol/L Fe + 0 mmol/L Si, (iv) Fe deficiency and Si application (10 Fe+Si): 10 μmol/L Fe + 1.5 mmol/L Si, (v) Fe deficiency treatment (1 Fe): 1 μmol/L Fe + 0 mmol/L Si, (vi) Fe deficiency and Si application (1 Fe+Si): 1 μmol/L Fe + 1.5 mmol/L Si. The concentration of 1 μM Fe was selected for further experiments based on the results of our preliminary experiments (100 μM Fe was the concentration of the normal nutrient solution, 1 μM and 10 μM Fe were the concentrations of Fe deficient nutrient solution) which eventually results in 4 treatments such as (i) Control (CK): 100 μmol/L Fe+ 0 mmol/L Si, (ii) Control and Si application (CK+Si): 100 μmol/L Fe + 1.5 mmol/L Si, (iii) Fe deficiency treatment (LF): 1 μmol/L Fe + 0 mmol/L Si, (iv) Fe deficiency and Si application (LF+Si): 1 μmol/L Fe + 1.5 mmol/L Si. Since K_2_SiO_3_ was used as the Si source, K^+^ introduced from K_2_SiO_3_ was subtracted from KNO_3_, and the loss of ${\text{NO}}_{3}^{-}$ was supplemented with diluted HNO_3_. During the experimental period, the nutrient solution was supplied with oxygen in an intermittent manner using an aeration pump, the pH of the nutrient solution was monitored daily and adjusted to 6.0 ± 0.2 using HNO_3_, the liquid level was replenished with distilled water every day and the nutrient solution was changed every 5 days. Biomass, photosynthetic fluorescence parameters, organic acid content, Fe content and transcriptomics were measured at 10 days of treatment, and other indexes such as antioxidant enzyme activity, leaf sucrose content and sucrose-related metabolic enzyme activity, etc. were measured in samples harvested at 5, 10, and 15 days of treatment.

### Transcriptomics analysis and quantative real-time PCR assay

At 10 d of treatment, leaf and root samples from the following 4 treatments were harvested and used for RNA-seq: (i) CK, (ii) CK+Si, (iii) LF, and (iv) LF+Si, and the prefixes L and R were used to denote the leaf and root tissues of tomato, respectively for data presentation. Three biological replicates were performed for each treatment, resulting in a total of 24 samples. Total RNA was extracted and purified using TRIzol reagent (Invitrogen, Carlsbad, CA, USA) according to the instruction of the manufacturer. NanoDrop ND-1000 (NanoDrop, Wilmington, DE, USA) was used to quantify the RNA quantity and purity of each sample. Bioanalyzer 2100 (Agilent, CA, USA) with RIN number >7.0, was used to assess the integrity of RNA and confirmed by electrophoresis with denaturing agarose gel. Two rounds of purification were used to purify Poly (A) RNA from 1 μg total RNA using Dynabeads Oligo (dT)25-61005 (Thermo Fisher, CA, USA). Then Magnesium RNA Fragmentation Module (NEB, cat.e6150, USA) was used to fragment the poly(A) RNA into small pieces under 94°C 5-7 min. To generate the cDNA, the cleaved RNA fragments were then reverse-transcribed by SuperScript™ II Reverse Transcriptase (Invitrogen, cat. 1896649, USA). The resulting cDNA were then used to synthesize U-labeled second-stranded DNAs with *E. coli* DNA polymerase I (NEB, cat.m0209, USA), RNase H (NEB, cat.m0297, USA) and dUTP Solution (Thermo Fisher, cat.R0133, USA). Then an A-base was added to the blunt ends of each strand which prepared them for ligation to the indexed adapters.The fragments were ligated to single- or dual-index adapters. After further processing, the ligated products were amplified with PCR under the following conditions: 3 min initial denaturation at 95°C; 8 cycles, 15 sec at 98°C (denaturation), 15 sec at 60°C (annealing), and 30 sec at 72°C (extension); and then 5 min at 72°C (final extension). The final cDNA library was 300 ± 50 bp in terms of the average insert size. At last, the 2×150bp paired-end sequencing (PE150) was performed on an Illumina Novaseq™ 6000 (LC-Bio Technology CO., Ltd., Hangzhou, China) following the recommended protocol. All raw sequencing data from the current study were deposited into the NCBI database under the accession number “PRJNA902026” (https://www.ncbi.nlm.nih.gov/bioproject/PRJNA902026), (Submitted on 15 November 2022). We used fastp software (https://github.com/OpenGene/fastp) to omit the reads that contained adaptor contamination, low quality bases and undetermined bases with default parameters. And fastp was also used to verify the sequence quality. To map reads to the reference genome of *S. lycopersicum L.* SL4.0, HISAT2 (https://ccb.jhu.edu/software/hisat2) was then used. Assembling of the mapped reads of each sample was performed using StringTie (https://ccb.jhu.edu/software/stringtie) with default parameters. Afterward, all transcriptomes from all samples were merged to reconstruct a comprehensive transcriptome using gffcompare (https://github.com/gpertea/gffcompare/). When the final transcriptome was generated, StringTie and was used to estimate the expression levels of all transcripts. StringTie analyzed the expression level for mRNAs by calculating FPKM (FPKM = [total_exon_fragments/mapped_reads(millions) × exon_length(kB)]). The differentially expressed mRNAs were selected with fold change > 2 or fold change < 0.5 and with parametric F-test comparing nested linear models (p-value < 0.05) by R package edgeR (https://bioconductor.org/packages/release/bioc/html/edgeR.html).

The real-time qPCR was performed using the gene-specific primers (**Table S1-S2**). Samples were added to the 96-well plate and then reacted in the Applied Biosystems Quant Studio 3 real-time fluorescence quantitative PCR instrument with the following conditions: Stage 1: pre-denatured, One cycle at 95°C for 30 s; Stage 2: PCR reaction, 40 cycles, 95°C for 10 s, 60°C for 30 s, and 72°C for 40 s.

### Measurements of biochemical and physiological parameters

The roots were cleaned and placed on the Epson Chops V800 Photo tray, scanned the root images and analyzed by the root analysis software WinRHIZO system. The chlorophyll and carotenoid contents were determined according to methods by Lichtenthaler and Wellburn (1983). The net photosynthetic rate (Pn) of functional leaves (the second fully expanded leaf at the growing point) of tomato seedlings on the 10th day of treatment was measured by portable photosynthetic apparatus (Li-6400; LI-COR, Lincoln, NE, USA). The maximum photochemical efficiency (Fv/Fm) of PSII was determined by Li-6400 portable photosynthetic apparatus fluorescence chamber after dark adaptation for 30 min. Then the leaves were activated under light for 1 h, and the effective photochemical quantum efficiency (Fv’/Fm’), actual photochemical quantum efficiency (ΦPSII), photosynthetic electron transfer rate (ETR) was, photochemical quenching coefficient (qP) and non-photochemical quenching coefficient (NPQ) of PSII under 800 μmol·m^-2^·s^-1^ activated light were determined. The Fe^3+^ reductase activity was determined according to 2,2 ‘-bipyridine-based methods. The Fe content was determined by flame spectrophotometry. The plant samples were digested by the H_2_SO_4_-H_2_O_2_ method and iron content was determined by atomic flame spectrophotometer (AA-3200). Leaf Fe distribution rate = leaf Fe content/(leaf Fe content + stem Fe content + root Fe content), the calculation of stem and root Fe distribution rate is the same as above.

The relative conductivity was measured with a conductivity meter. MDA content was determined by the method of Heath and Packer (1968). H_2_O_2_ content was determined by the method of Willekens et al. (1997). The superoxide anion content was determined by the method described previously (Zhang et al., 2019a). Antioxidant enzyme activity was determined as described by Sheteiwy et al. (2017).

The sucrose content was determined by the hydrochloric acid-resorcinol method. The activities of sucrose neutral invertase (NI), acid invertase (AI), sucrose synthase (SS) and sucrose phosphate synthase (SPS) were determined using the corresponding enzyme activity assay kits (Beijing Solarbio Science & Technology Co., Ltd., Beijing, China). The content of organic acids was determined by high-performance liquid chromatography as previously described (Shi et al., 2022).

### Statistical analysis

All physiological data were checked for statistical significance using ANOVA and presented as the mean ± standard deviation (SD) of 3 biological replicates. Duncan’s multiple range test was applied to compare the means at the *P*<0.05 level in SPSS (version-22.0).

## Results

### Silicon alleviated Fe deficiency-induced growth inhibition

To elucidate the effect of Fe deficiency on tomato plants, the growth of tomato seedlings under Fe deficiency was assessed. As shown in **Table 1** and **Supplementary Figure 1**, Fe deficiency repressed biomass and significantly changed different root features. The superoxide anion However, the application of Si alleviated the growth inhibition caused by Fe deficiency in tomato seedlings. For instance, LF significantly decreased shoot biomass by 56.25% compared with CK; however, LF+Si significantly increased shoot biomass by 69.64% compared with LF (**Supplementary Figure 1**). Moreover, Si application mitigated the reduction in root length and root surface area caused by Fe deficiency (**Table 1**). More precisely, LF significantly decreased root length by 54.65% compared with CK; however, LF+Si significantly increased root length by 36.80% compared with LF.

**Table 1:** Silicon improved different root features in tomato seedlings under Fe deficiency.

Treatments	Total root length (cm)	Total root surface area (cm^2^)	Total root volume (cm^3^)
CK	377.51 ± 30.85 b	44.15 ± 2.52 bc	1.03 ± 0.09 a
CK+Si	434.68 ± 22.93 a	69.46 ± 3.62 a	0.95 ± 0.03 a
LF	171.19 ± 0.45 d	39.56 ± 0.97 c	0.54 ± 0.01 c
LF+Si	234.18 ± 6.96 c	44.64 ± 3.36 b	0.73 ± 0.02 b

Control (CK): 100 μmol/L Fe+ 0 mmol/L Si, Control and Si application (CK+Si): 100 μmol/L Fe + 1.5 mmol/L Si, Fe deficiency treatment (LF): 1 μmol/L Fe + 0 mmol/L Si, and Fe deficiency and Si application (LF+Si): 1 μmol/L Fe + 1.5 mmol/L Si. Different letters in the same column indicate statistically significant differences (P<0.05).

### Differentially expressed gene analyses

**Figure 1:**
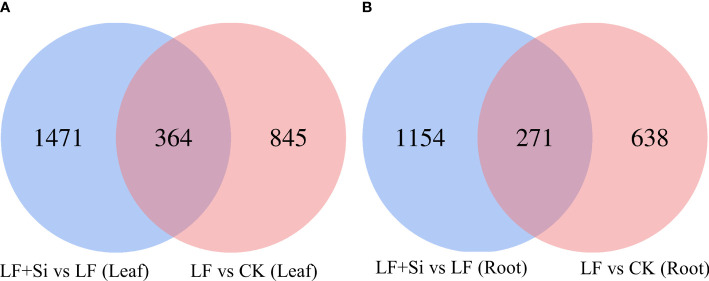
Venn diagram of differentially expressed genes in tomato seedlings as influenced by Fe deficiency and Si application. **(A)** The differentially expressed genes in leaves. **(B)** The differentially expressed genes in roots. Control (CK): 100 μmol/L Fe+ 0 mmol/L Si, Fe deficiency treatment (LF): 1 μmol/L Fe + 0 mmol/L Si, and Fe deficiency and Si application (LF+Si): 1 μmol/L Fe + 1.5 mmol/L Si.

Compared with the control, 1209 and 909 differentially expressed genes (DEGs) were found in the leaf and root, respectively, under Fe deficiency (**Figure 1**). Compared with Fe deficiency, 1835 and 1425 DEGs were found in the leaf and root when treated with Si (**Figure 1**).

**Figure 2:**
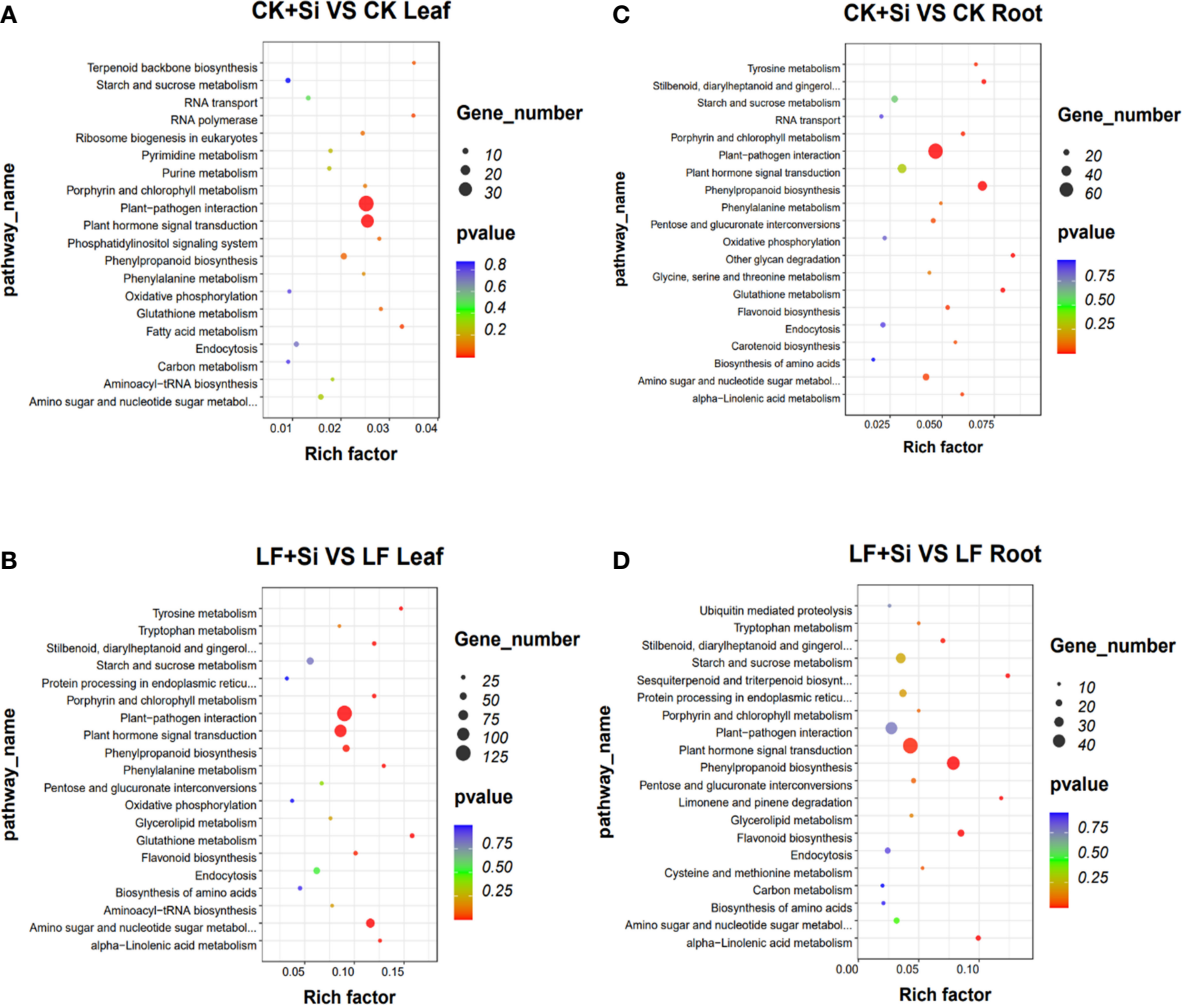
The KEGG enrichment analysis of differentially expressed genes. **(A, B)** The KEGG enrichment analysis in leaves. **(C, D)** The KEGG enrichment analysis in roots.

KEGG pathway enrichment analysis showed that in leaves, glutathione metabolism was mainly related to Fe deficiency stress and Si application, followed by biosynthesis of amino acids and plant hormone signal transduction (**Figures 2A, B**). However, in roots, it is mostly correlated with flavonoid biosynthesis, followed by nitrogen metabolism, amino sugar and nucleotide sugar metabolism (**Figures 2C, D**). In the KEGG pathway, which was significantly enriched in the leaf, we focused on porphyrin and chlorophyll metabolism and photosynthesis-antenna proteins.

**Figure 3:**
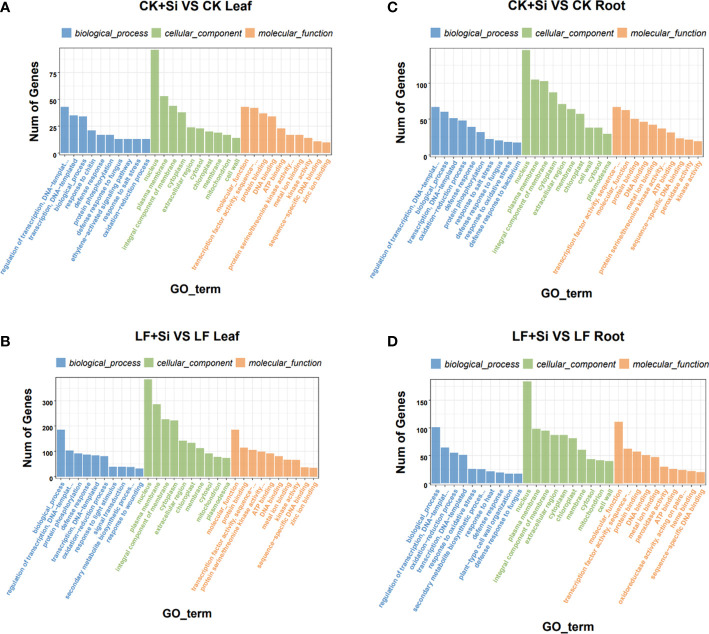
The GO enrichment analysis of differentially expressed genes. **(A, B)** The GO enrichment analysis in leaves. **(C, D)** The GO enrichment analysis in roots.

GO enrichment analysis showed that response to UV was most related to Fe deficiency and Si application in the leaf, followed by response to salicylic acid and protochlorophyllide reductase activity (**Figures 3A, B**). However, in roots, it is mostly correlated with L-leucine transaminase activity, followed by the anchored component of plasma membrane, plant−type cell wall organization or biogenesis (**Figures 3C, D**). In the GO functional enrichment analysis results, we focused on peroxidase activity, ion transport and ferric-chelate reductase activity.

### Silicon improved photosynthesis under Fe deficiency

**Figure 4:**
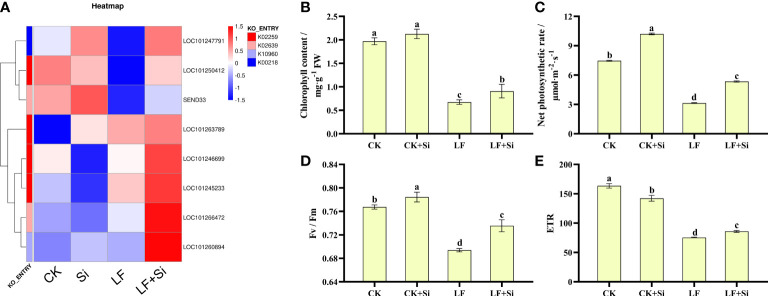
Response of photosynthetic capacity of tomato to silicon application under Fe deficiency. **(A)** Heat map of photosynthesis-related differentially expressed genes. **(B)** Total chlorophyll content. **(C)** Net photosynthetic rate. **(D)** Maximum photochemical efficiency of PS II (Fv/Fm). **(E)** Photosynthetic electron transport rate (ETR). Each data point represents the mean of three independent biological replicates (mean ± SD). Different letters above the bars indicate statistically significant differences (*P* < 0.05).

Photosynthetic pigments are one of the most important indicators of the photosynthetic capacity of plants and can be used together with photosynthetic parameters to reflect changes in photosynthetic efficiency and assimilation capacity. After enrichment analysis of differentially expressed gene KEGG, DEGs in porphyrin and chlorophyll metabolism and photosynthesis-antenna protein pathways were selected to make heat maps (**Figure 4A**). We found that the Si application under Fe deficiency mainly upregulated *SEND33*, LOC101263789, LOCLOC101260894, and LOC101266472. *SEND33* encodes Solanum lycopersicum ferredoxin-I, which is also the nuclear gene for chloroplast products. As shown in **Figure 4**, chlorophyll contents were significantly reduced under Fe deficiency. However, the application of Si increased chlorophyll contents under Fe deficiency. LF significantly decreased chlorophyll contents by 65.99% compared with CK; however, LF+Si significantly increased chlorophyll contents by 35.82% compared with LF. As shown in **Figures 4B**, Fe deficiency reduced Pn, Fv/Fm and ETR. However, the application of Si increased Pn, Fv/Fm and ETR under Fe deficiency (**Figures 4C, D, E**,. For instance, LF significantly decreased net photosynthetic rate by 57.91% compared with CK; however, LF+Si significantly increased net photosynthetic rate by 70.06% compared with LF. Moreover, the values of ΦPSII, qP and NPQ decreased significantly under Fe deficiency, but the addition of Si increased ΦPSII and NPQ (**Table S3**). These results indicate that Fe deficiency reduced the photosynthetic capacity of tomato, while Si improved photosynthetic performance.

### Silicon alleviated Fe deficiency-induced stress damage

**Figure 5:**
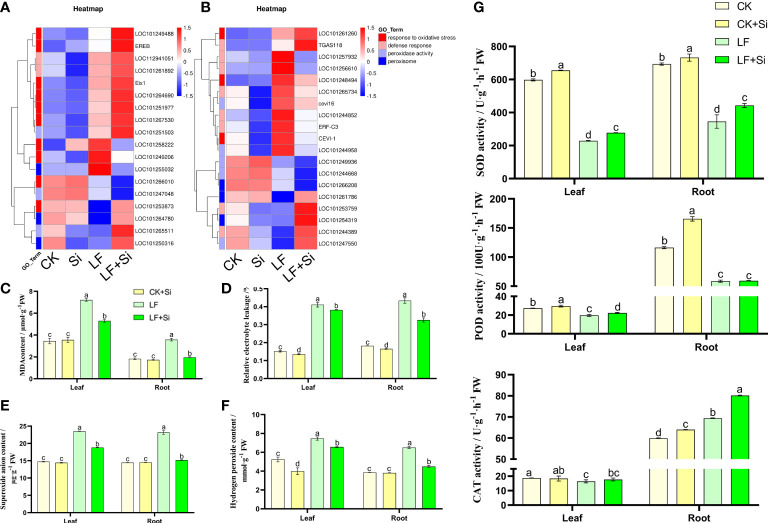
Effects of silicon application on antioxidant defense in tomato under Fe deficiency. **(A)** Heat map of antioxidant-related differentially expressed genes in the leaf. **(B)** Heat map of antioxidant-related differentially expressed genes in the root. **(C)** malondialhyde (MDA) content. **(D)** Relative electrolyte leakage. **(E)** Hydrogen peroxide content. **(F)** Superoxide anion content. **(G)** Antioxidant enzyme activity. Each data point represents the mean of three independent biological replicates (mean ± SD). Different letters above the bars indicate statistically significant differences (*P*<0.05).

Oxidative stress is intensified under Fe deficiency. Through GO functional gene annotation, we found that part of DEGs was enriched in the response to oxidative stress (GO:0006979), defense response (GO:0006952), peroxidase activity (GO:0004601) and peroxisome (GO:0005777) function (**Figure 5A**). Moreover, we found that Si application under Fe deficiency mainly upregulated *EREB* and *Eix1* in leaves (**Figure 5A**), and upregulated *TGAS118* in root*s. EREB* encodes Solanum lycopersicum ethylene responsive element binding protein. As shown in **Figures 5C, D**, Fe deficiency resulted in increased electrolyte permeability and MDA content in tomato seedlings. However, Si application reduced electrolyte permeability and MDA content. Compared with CK, MDA content in leaves and roots of LF increased 1.08 and 0.96 times, respectively. The MDA content of LF+Si significantly decreased by 26.44% and 45.14% compared with LF in leaves and roots, respectively. Likewise, compared with CK, H_2_O_2_ content in leaves and roots of LF increased by 42.37% and 69.18%, respectively. However, compared with LF, the H_2_O_2_ content in LF+Si significantly decreased by 11.97% and 31.11% in leaves and roots, respectively. The excessive accumulation of ROS disrupted the cellular enzymatic antioxidant defense system (**Figures 5E, F**). However, Si application alleviated the accumulation of ROS and MDA under Fe deficiency stress, by enhancing the efficiency of the enzymatic antioxidant system such as the activity of SOD in leaves and roots (**Figure 5**).

### Silicon enhanced organic acid metabolism under Fe deficiency

**Figure 6:**
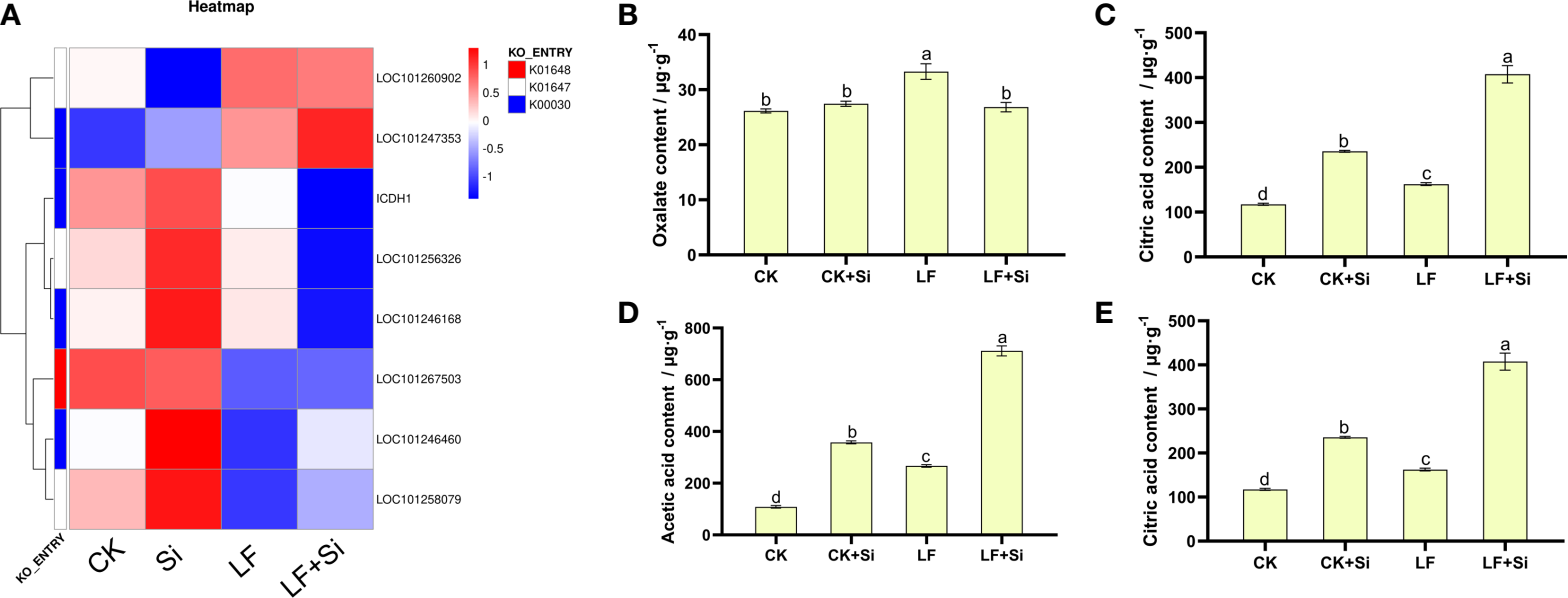
Effects of silicon application on organic acid metabolism in tomato roots under Fe deficiency. **(A)** Heat map of related differentially expressed genes. **(B)** Oxalate content. **(C)** Malic acid content. **(D)** Acetic acid content. **(E)** Citric acid content. Each data point represents the mean of three independent biological replicates (mean ± SD). Different letters above the bars indicate statistically significant differences (*P*<0.05).

Organic acid metabolism is not only regarded as one of the necessary metabolic processes for plant growth, but also one of the intermediate links for plant iron absorption. Therefore, the content of organic acids in roots, an important organ for absorbing nutrients, was determined. By focusing on the Citrate cycle (ko00020), we found that differentially expressed genes are closely related to citric acid metabolism. We found that the Si application under Fe deficiency upregulated LOC101247353 and LOC101258079, and downregulated *ICDH1* (**Figure 6**). *ICDH1* encodes Solanum lycopersicum isocitrate dehydrogenase [NAD] regulatory subunit 1. As shown in **Figure 6**, the content of oxalic acid, malic acid, acetic acid and citric acid contents in tomato roots increased under Fe deficiency. However, increased application of Si further increased the organic acid content (**Figure 6**). Compared with CK, malic acid, acetic acid and citric acid contents of LF increased by 105.21%, 145.54% and 38.39%, respectively. Interestingly, compared with LF, the malic acid, acetic acid and citric acid contents of LF+Si significantly increased by 53.34%, 166.75% and 150.88%, respectively.

### Silicon enhanced sugar metabolism under Fe deficiency

**Figure 7:**
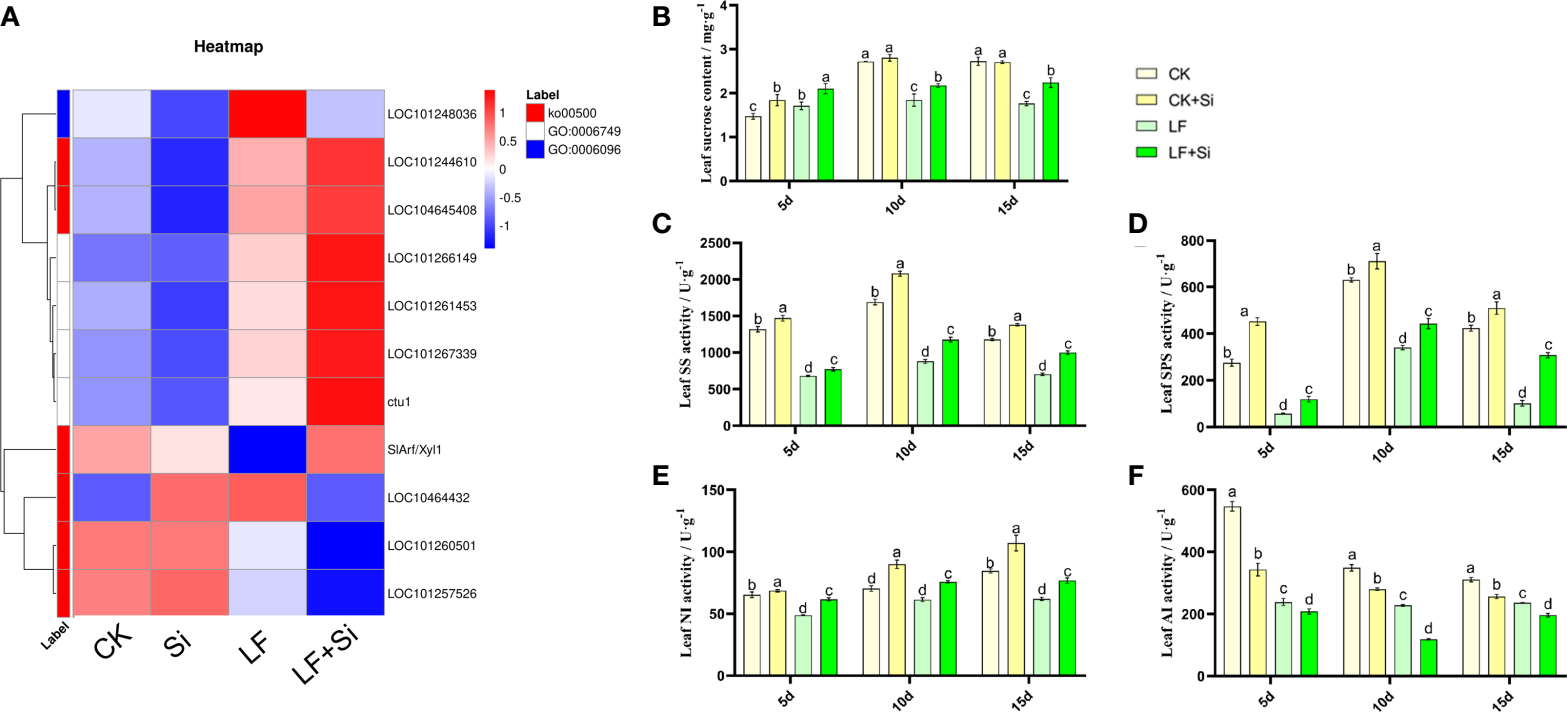
Response of sucrose metabolism of tomato leaves to silicon application under Fe deficiency. **(A)** Heat map of related differentially expressed genes. **(B)** Leaf sucrose content. **(C)** Leaf SS activity. **(D)** Leaf SPS activity. **(E)** Leaf NI activity. **(F)** Leaf AI activity. Each data point represents the mean of three independent biological replicates (mean ± SD). Different letters above the bars indicate statistically significant differences (*P*<0.05).

To further investigate the effects of nutrient metabolism under Fe deficiency, we measured the relevant indicators of sucrose metabolism in tomato leaves. By focusing on Starch and sucrose metabolism (ko00500), we found that Si application under Fe deficiency upregulated *ctu1* and *SlArf/Xyl1*, downregulated LOC101257526 and LOC101260501 (**Figure 7**). *ctu1* encodes solanum lycopersicum glutation-S-transferase. As shown in **Figure 7**, the sucrose content of the leaves showed an increase at 5 and 10 d and a decrease at 15 d under Fe deficiency. LF significantly decreased sucrose content by 35.29% compared with CK at 15 d. However, LF+Si significantly increased sucrose content by 27.00% compared with LF at 15 d (**Figure 7**). Subsequently, the activities of key enzymes for synthesis and conversion in sucrose metabolism were measured. As shown in **Figures 7C, D**, SS and SPS activities followed the same trend as sucrose content, showing an increase at 5 and 10 d and a decrease at 15 d. NI activity decreased under low iron stress, increased in the Si addition, and increased progressively over time in all treatments (**Figure 7**). AI activity decreased progressively over time and, Si addition also reduced AI activity (**Figure 7**).

### Silicon enhanced iron absorption capacity under Fe deficiency

**Figure 8:**
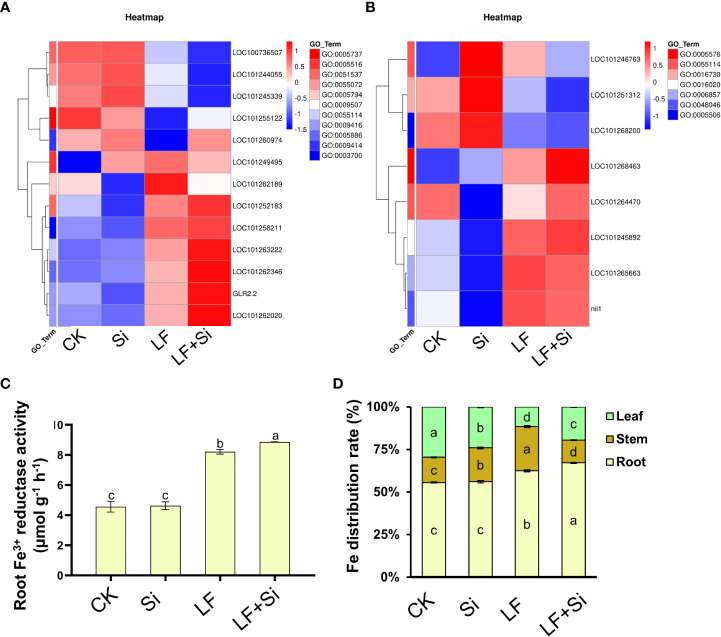
Effects of silicon application on absorption capacity of Fe under Fe deficiency in tomato. **(A)** Heat map of related leaf differentially expressed genes. **(B)** Heat map of related root differential genes. **(C)** Root Fe^3+^ reductase activity. **(D)** Fe distribution rate (%) in tomato plants. Each data point represents the mean of three independent biological replicates (mean ± SD). Different letters above the bars indicate statistically significant differences (*P*<0.05).

The root system is main organ for absorbing iron. Through GO functional gene annotation, we found that part of DEGs was enriched in the cellular response to iron ion (GO:0071281), iron ion homeostasis (GO:0055072), and iron ion binding (GO:0005506) function that we were interested in (**Figure 8A**). Moreover, we found that Si application under Fe deficiency mainly upregulated *GLR2.2* in leaves, and upregulated LOC101268463 in roots. *GLR2.2* encodes solanum lycopersicum glutamate receptor 2.2. The reduction process involving Fe^3+^ reductase is the rate-limiting process by which plants acquire Fe from the soil and can reflect the rate of Fe uptake by plants. As shown in **Figure 8**, the Fe^3+^ reductase activity was increased by 79.93% under Fe deficiency compared with CK, and Si application further enhanced root Fe^3+^ reductase activity. The accumulation of Fe in tomato plants changed under Fe deficiency. For instance, compared with CK, LF reduced the Fe content in leaves by 54.97%; however, LF+Si increased the Fe content in leaves by 31.86% compared with LF (**Supplementary Figure 2**). Moreover, we found that the application of Si under Fe deficiency significantly promoted the distribution of Fe to leaves and roots in tomato seedlings, suggesting a role for Si in modulating the transport of Fe from root and stem to leaf under Fe deficiency (**Figure 8**). Consistent with this, the transcript levels of iron transporters such as *FRD3, IRT2* and *FRO6* were significantly uprgulated by LF+Si compared with LF only treatment (**Figure S3**).

**Figure 9:**
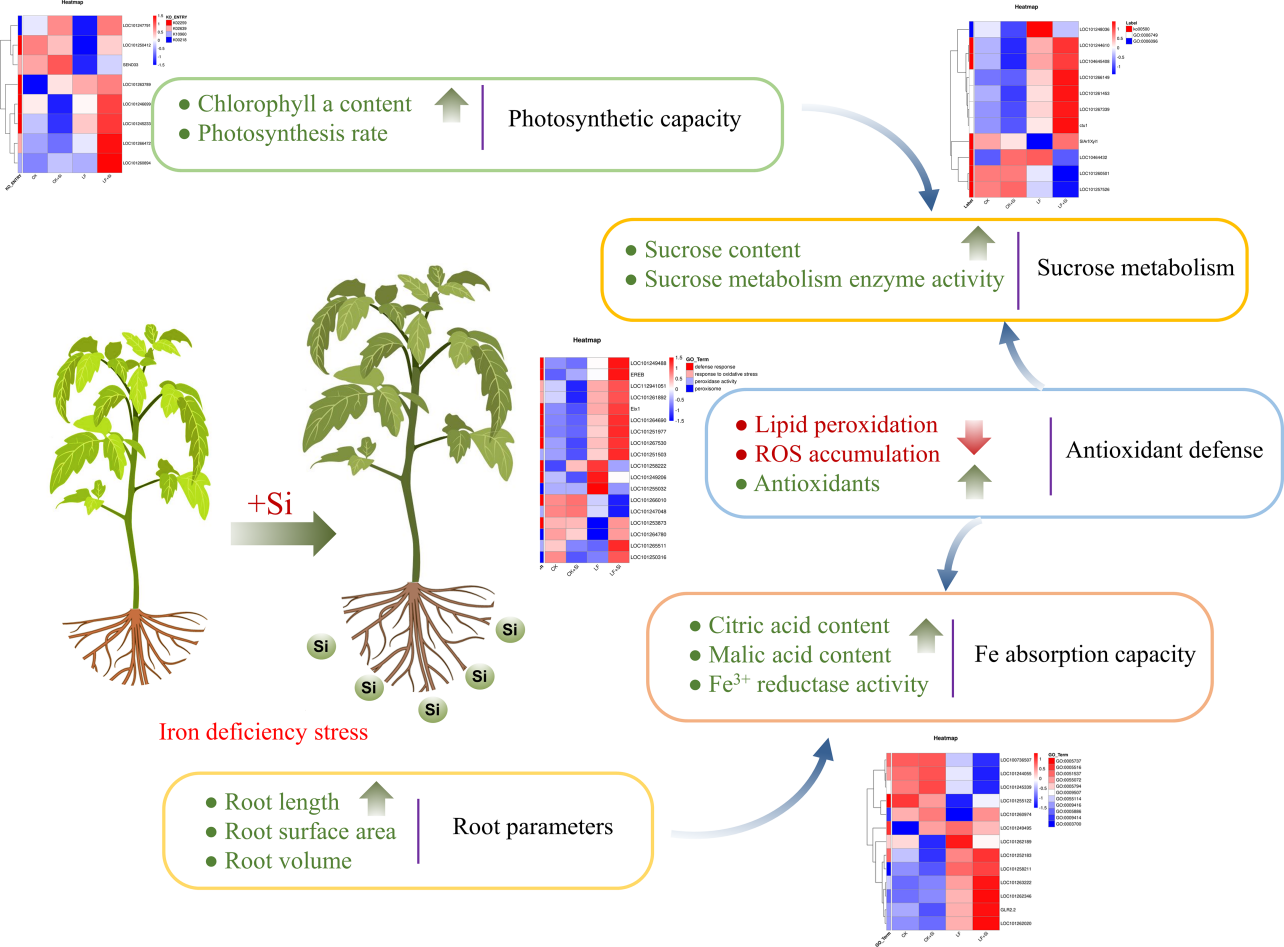
A working model of the effect of Si application on tomato under Fe deficiency.

Taken together, we found that Si application under Fe deficiency affected the photosynthetic capacity, sucrose metabolism, antioxidant capacity, Fe absorption capacity and root growth of tomato (**Figure 9**). Fe deficiency firstly mediated a strong oxidative stress response in tomato, while Si application effectively maintained the stability of the metabolic environment by improving the activity of antioxidant enzymes to remove excess ROS. Si application significantly increased chlorophyll biosynthesis and brought more photosynthetic products, thus promoting the transformation of sugar metabolism in leaves. In roots, Si application increased the root surface area and maintained the dynamic balance of ROS, which together improved the Fe absorption capacity of tomato.

## Discussion

Iron deficiency is a major handicap for crop production in many soils worldwide. In the current study, we provided physiological, biochemical and molecular evidence that Si supply could alleviate Fe deficiency and revealed critical mechanisms through which Si improved tolerance to Fe deficiency in tomato. Transcriptomic data combined with physiological analyses provide a novel characterization of the key traits and stress tolerance associated with the adaptation of tomato plants to Fe deficiency. Traits associated with changes in gene expression towards enhanced tolerance to Fe deficiency can be used to improve protected tomato cultivation.

Biomass usually directly reflects the difference in horticultural plant growth and response to the environment, and Fe deficiency significantly decreased plant biomass accumulation as reflected by the decline in the fresh weight of tomato seedlings as well as the changes in root morphology that directly affects the ability of roots to absorb and transport water and nutrients (Zhan et al., 2015). In order to adapt to the Fe deficiency, roots undergo two changes: one is to inhibit the growth of taproots to reduce the consumption of photosynthates; the other is to increase the number of lateral roots to enhance nutrient absorption capacity (Debona et al., 2017; Zhang et al., 2019b; Chao and Chao, 2022). Si application improved the tolerance of tomato to Fe deficiency by improving root architecture (**Table 1**). Si application promoted lateral root initiation, and such root architecture change was potentially beneficial to increase tomato root nutrient absorption area (Wang et al., 2013).

Photosynthesis is very sensitive to environmental stress, nonetheless, Si has been found to improve photosynthetic gas exchange in a variety of plants, including tomato, sorghum, pumpkin and tobacco (Haghighi and Pessarakli, 2013; Hajiboland and Cheraghvareh, 2014; Li et al., 2015; Siddiqui et al., 2015). The KEGG pathway analysis of differentially expressed genes in leaves revealed 8 DEGs in porphyrin and chlorophyll metabolism in LF+Si treatment compared to LF treatment in leaves (**Figure 4**). Notably, Si application protected the integrity of the chloroplast structure and also increased the chlorophyll content, leading to improved absorption and transfer of light energy (Zhu and Gong, 2013).

Transcriptome analysis showed that Si application under Fe deficiency increased the activity of Fe chelate reductase and the expression level of ferridoxin in tomato roots. Ferridoxin is a small molecule protein with an electron transport function, and its content can reflect the efficiency of electron transfer, thus affecting photosynthesis (Mazor et al., 2015). In addition, the formation of silicified cells due to Si application can increase the net photosynthetic rate of the plant (Liang et al., 2003). The high photosynthetic rate increases the accumulation of photosynthetic products of the plant and accelerates the process of carbon and nitrogen metabolism, thus increasing biomass accumulation (Noshi et al., 2016; Ahmad et al., 2021).

Chlorophyll fluorescence parameters are used to reflect the various reactive processes in plant photosynthesis under abiotic stress (Zai et al., 2012; Guo and Tan, 2015). Under Fe deficiency, the values of ΦPSII, ETR and qP decreased significantly, but increased after the addition of Si (**Supplementary Table 3**), indicating that the Si application effectively increased the development and activity of PSII reaction center, which was conducive to using more energy for PSII electron transfer and improving the efficiency of photosynthetic pigments in converting light energy into chemical energy. NPQ decreased under Fe deficiency and Si application increased NPQ, indicating that Fe deficiency damaged the photoprotective system, limiting the dissipation of excess light energy and increasing the risk of damage to the photosystem, while Si application effectively protected the photoprotective system and reduced the damage to the photosystem from the accumulation of excess light energy (Debona et al., 2017; Hussain et al., 2021).

The generation and clearance of ROS in plants are in a state of dynamic balance to maintain normal growth and metabolism (Mittler et al., 2022). Under Fe deficiency, ROS such as H_2_O_2_, and superoxide anion accumulate in large quantities, causing oxidative damage to proteins, nucleic acids and lipids and thus destroying the normal growth and metabolism of plants (Puyang et al., 2015; Zhu et al., 2015). Plants deploy their antioxidant defense by increasing the activity of antioxidant enzymes such as SOD, POD and CAT and by accumulating antioxidant substances such as ascorbic acid and glutathione (Ahmad et al., 2019; Zhang et al., 2019a; Ahanger et al., 2020). Transcriptomic analysis showed that Si treatment significantly increased the expression peroxidase *cevi16* under Fe deficiency (**Figure 5**). Transcriptome data and physiological indicators suggest that Si alleviates the membrane lipid peroxidation induced by Fe deficiency by enhancing the activity of antioxidant enzymes in plants, reduces the accumulation of ROS in plants, and protects the integrity of cell membranes, thus improving the tolerance of plants to Fe deficiency (Zhu et al., 2015).

Transcriptome analysis showed that citrate synthase and malase genes were up-regulated in leaves under Fe deficiency, and the up-regulated genes mainly encoded citrate synthase and malase in roots under Fe deficiency combined with Si application (**Figure 6**). It is possible that Si promoted the secretion of organic acids and alleviated the Fe deficiency by up-regulating the expression of genes encoding enzymes related to organic acid synthesis. In dicotyledon plants, only divalent Fe can be transported, generally in the chelated form with citric acid or malic acid. Both malic acid and citric acid are involved in the chelation of Fe in the xylem. The Fe-citric acid complex is involved in long-distance Fe transport in plant xylem (Bityutskii et al., 2018). Pavlovic et al. (2013) found that the Si-mediated alleviatory effects on Fe deficiency included Fe activation and Fe absorption in root exoplasms (Pavlovic et al., 2013). The increase in Fe binding transport compounds (such as citric acid) is the main mechanism of Si-induced alleviation of Fe deficiency in cucumber plants (Bityutskii et al., 2014). Moreover, the accumulation of organic compounds associated with Fe absorption and transport in roots and exudation of root exudates largely increase Fe availability due to Fe^3+^ chelation and reduction (Martínez-Cuenca et al., 2013). Bityutskii et al. (2017) showed that the content of citric acid was very low under Fe deficiency at pH 4, indicating that organic acids did not respond to Fe deficiency under acidic conditions (Bityutskii et al., 2017). At pH 6, the concentrations of several organic acids, including citric acid, succinic acid, fumaric acid and gluconic acid increased under Fe deficiency (Bityutskii et al., 2017). Although citric acid is not necessarily accumulated in strawberry roots under Fe deficiency, it is released with root exudates (Valentinuzzi et al., 2015). Therefore, the increase in root organic acid content induced by Si application is important in promoting the long-distance transport of Fe (Bityutskii et al., 2017).

The metabolism of starch and sucrose is one of the important mechanisms of plant response to Fe deficiency. During Fe deficiency, the expression of genes related to glycolytic pathway was significantly up-regulated, and Fe deficiency significantly affected the sugar metabolism in Malus chinensis (Hu et al., 2018). In this study, GO functional enrichment analysis of up-regulated differentially expressed genes showed that Si application under Fe deficiency up-regulated the expression of genes related to starch and sucrose metabolism. The results showed that the synthesis, utilization and distribution of starch and sucrose in tomato leaves and roots were affected by Fe deficiency, and the regulation of sugar metabolism was a way of tomato plant response to Fe deficiency. Under abiotic stress, a large amount of soluble sugar is accumulated, which further inhibits photosynthesis and slows down plant growth (Erban et al., 2015; Wei et al., 2015) and sucrose is decomposed into hexose for use by leaves to maintain the normal growth of plants (Hütsch et al., 2014). The results showed that in the early stage of Fe deficiency, sucrose accumulation increased in response to Fe deficiency, which could play a role in osmotic regulation. In order to further explore the mechanism of alterations in sucrose content, we further detected the activity of sucrose metabolism enzymes. The results showed that the decrease in sucrose content in leaves under Fe deficiency was due to the decrease in SS, SPS, NI and AI activities (**Figure 7**). However, with the increase of stress duration, the utilization rate of sugar in plant tissues decreased, which would change the source-sink relationship and lead to the failure of timely transportation of photosynthates, resulting in feedback inhibition (Luo et al., 2021).

The reduction of Fe^3+^ by Fe^3+^ reductase in roots of the Strategy I plant, is the rate-limiting step of Fe absorption (Connolly et al., 2003). The Strategy I plants increase root H^+^-ATPase and root Fe^3+^ reductase activities under Fe deficiency, release a large amount of H^+^, reduce the rhizosphere pH, and then promote the activation and absorption of Fe (Song et al., 2018). Our results showed that Si application under Fe deficiency promoted the expression of *IRT2* and *FRO6* in roots (**Supplementary Figure S2**) and increased the activity of Fe^3+^ reductase (**Figure 8**). Si application promoted Fe absorption and improved Fe transport efficiency from roots to leaves by Fe^3+^ reductase activity (Bityutskii et al., 2017). In addition, Si affects the gene expression in the synthesis of Fe transport-related compounds, resulting in an increased accumulation of organic acids and phenols, thus increasing the availability of Fe in roots (Carrasco-Gil et al., 2018).

## Conclusions

In summary, Fe deficiency inhibits tomato plant growth by inducing oxidative stress, and inhibiting photosynthesis and glucose metabolism in tomato seedlings. Transcriptomics analysis coupled with biochemical assays indicated that Si modulated the response of plants to Fe deficiency by regulating antioxidant response, and the expression of related genes involved in carbohydrate metabolism. Moreover, Si application protected photosynthesis possibly by improving the antioxidant defense, and maintained Fe-related physiological metabolism by promoting Fe distribution in tomato leaves and roots under Fe deficiency. The study provides an important reference for the underlying mechanism of Si-induced tolerance to low Fe stress and the potential utilization of Si fertilizer to improve the growth traits of tomato plants under Fe deficiency.

## Data availability statement

The data presented in the study are deposited in the NCBI repository (https://www.ncbi.nlm.nih.gov/bioproject/PRJNA902026), accession number: PRJNA902026.

## Author contributions

YS: Conceptualization, Methodology, Formal analysis,Investigation, Writing—original draft. SG: Formal analysis, Investigation, Writing—original draft. XZ: Formal analysis, Investigation. MX: Methodology, Investigation. JX: Formal analysis, Investigation. GX: Funding acquisition, Project administration. YZ: Conceptualization, Supervision, Resources, Writing—original draft, Project administration. GA: Conceptualization, Writing—review and editing, Project administration. All authors contributed to the article and approved the submitted version.
