# Supplementary material for: Comparative physiological and transcriptomics analysis revealed crucial mechanisms of silicon-mediated tolerance to iron deficiency in tomato
Source: Front Plant Sci. 2022 Dec 21;13:1094451. doi: 10.3389/fpls.2022.1094451 (PMC9811145; doi:10.3389/fpls.2022.1094451)
Supplement: Supplementary file 1 [file DataSheet_1.pdf]

Supplementary materials

Comparative physiological and transcriptomics analysis revealed crucial mechanisms of silicon-mediated tolerance to Fe deficiency in tomato

Yu Shi[1±], Shuxun Guo[1±], Xin Zhao[1], Mengzhu Xu[1], Jin Xu[1], Guoming Xing[1], Yi Zhang[1,*] and Golam Jalal Ahammed[2,*]

[1]College of Horticulture, Shanxi Agricultural University, Taigu, Shanxi, China.

[2]College of Horticulture and Plant Protection, Henan University of Science and Technology, Luoyang, Henan, China.

[*]Correspondence: Yi Zhang (harmony1228@163.com) or Golam Jalal Ahammed (ahammed@haust.edu.cn)

[±]These authors contributed equally to this work: Yu Shi, Shuxun Guo

Supplementary Table 1. qRT-PCR test reaction system

Reactant	Concentration	Volume（μL）
ChamQ Universal SYBR Color qPCR Master Mix	2X	10
Primers-F	10μM	0.4
Primers-R	10μM	0.4
Template (cDNA)		2

Supplementary Table 2. Primers used for qRT-PCR

Gene	Accession No.	Forward primer	Reverse primer
Actin	*Solyc03g078400*	ACCACTGAGCACAATGTTACCG	GTCCTCTTCCAGCCATCCA
FRD3	*Solyc11g013440*	TCCATTGGGGTGTCAGTGGTG	CTGCGTTGTGCCTAATCGTGC
IRT2	*Solyc02g069190*	AATCCAGAAACTGGTGGTGCTG	GAAAAGTATACACGATTACAATTTTGC
FRO6	*Solyc01g102610*	AACACCCAAAGCAATGCACC	GCCCCTTGTACCACCAGTAA

Supplementary Table 3. Effects of silicon on chlorophyll fluorescence parameters of tomato leaves

Treatment	Fv'/Fm'	Φ_{PSII}	qP	NPQ
CK	0.57±0.00 b	0.37±0.01 a	0.64±0.00 a	2.42±0.07 b
CK+Si	0.63±0.00 a	0.33±0.01 b	0.52±0.02 b	2.68±0.01 a
LF	0.50±0.01 c	0.17±0.00 d	0.32±0.02 c	2.18±0.11 c
LF+Si	0.58±0.00 b	0.20±0.00 c	0.34±0.00 c	2.37±0.01 b

Control (CK): 100 μmol/L Fe+ 0 mmol/L Si, Control and Si application (CK+Si): 100 μmol/L Fe + 1.5 mmol/L Si, Fe deficiency treatment (LF): 1 μmol/L Fe + 0 mmol/L Si, and Fe deficiency and Si application (LF+Si): 1 μmol/L Fe + 1.5 mmol/L Si. Each data point represents the mean of three independent biological replicates (mean ± SD). Different letters in the same column indicate statistically significant differences (P<0.05).

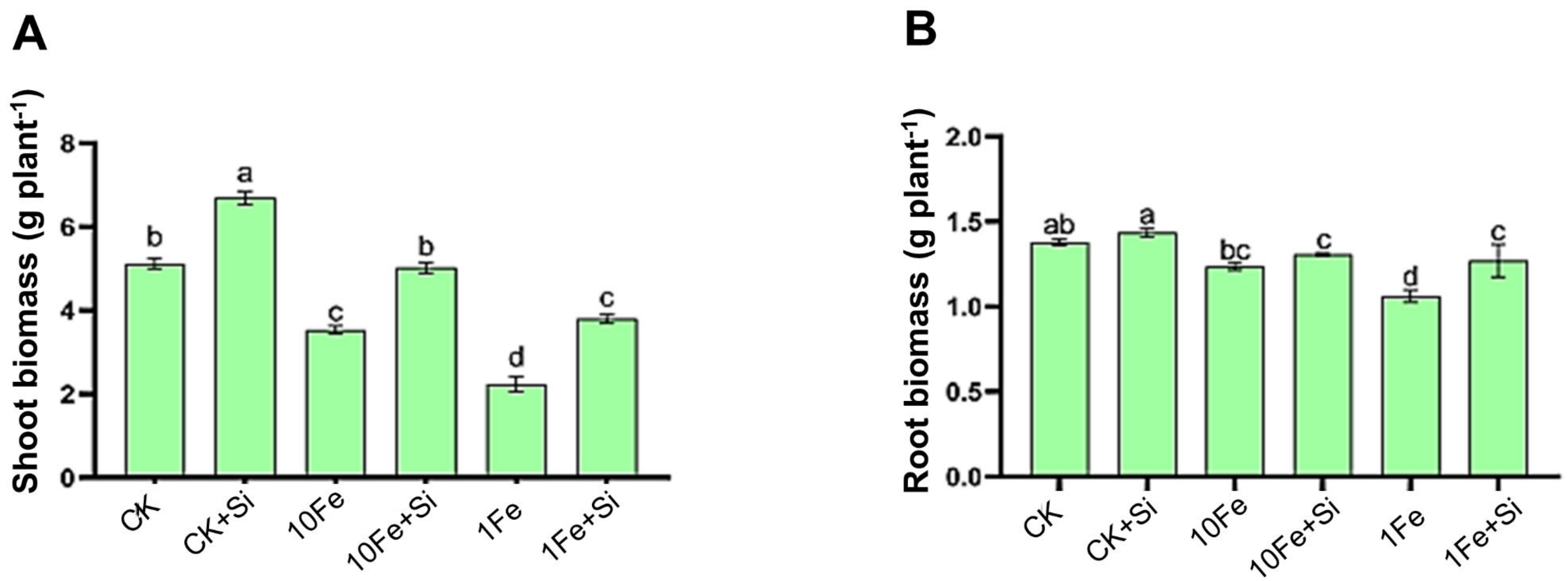

Supplementary Figure 1 Silicon (Si) alleviated growth inhibition in tomato seedlings under Fe deficiency. (**A**) Shoot biomass. (**B**) Root biomass. Control (CK): 100 μmol/L Fe+ 0 mmol/L Si, Control and Si application (CK+Si): 100 μmol/L Fe + 1.5 mmol/L Si, moderate Fe deficiency treatment (10Fe): 10 μmol/L Fe + 0 mmol/L Si, moderate Fe deficiency and Si application (10Fe+Si): 10 μmol/L Fe + 1.5 mmol/L Si, high Fe deficiency treatment (1Fe): 1 μmol/L Fe + 0 mmol/L Si, and high Fe deficiency and Si application (1Fe+Si): 1 μmol/L Fe + 1.5 mmol/L Si. Each data point represents the mean of three independent biological replicates (mean ± SD). Different letters above the bars indicate statistically significant differences (P<0.05).

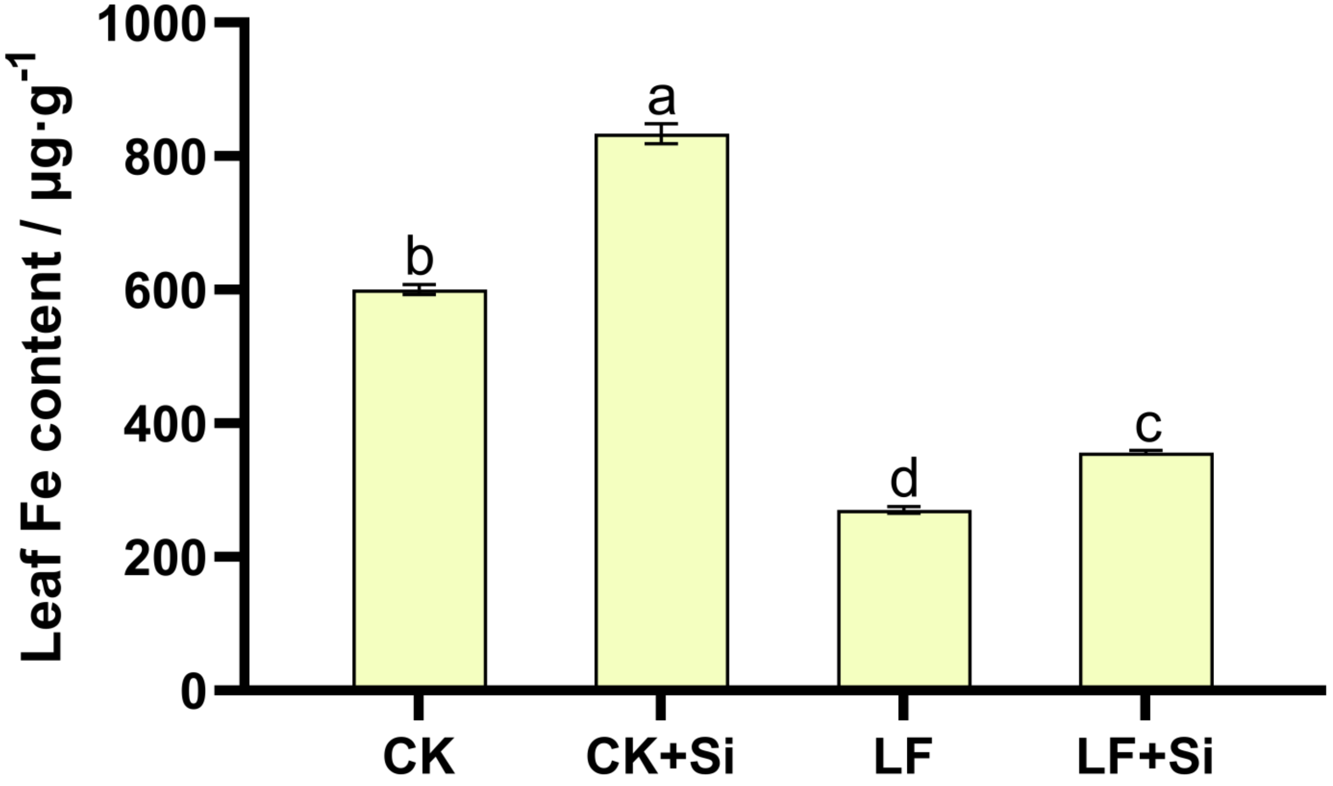

Supplementary Figure 2 Effects of exogenous silicon application on leaf Fe content under Fe deficiency. Each data point represents the mean of three independent biological replicates (mean ± SD). Different letters above the bars indicate statistically significant differences (P<0.05). Control (CK): 100 μmol/L Fe+ 0 mmol/L Si, Control and Si application (CK+Si): 100 μmol/L Fe + 1.5 mmol/L Si, Fe deficiency treatment (LF): 1 μmol/L Fe + 0 mmol/L Si, and Fe deficiency and Si application (LF+Si): 1 μmol/L Fe + 1.5 mmol/L Si.

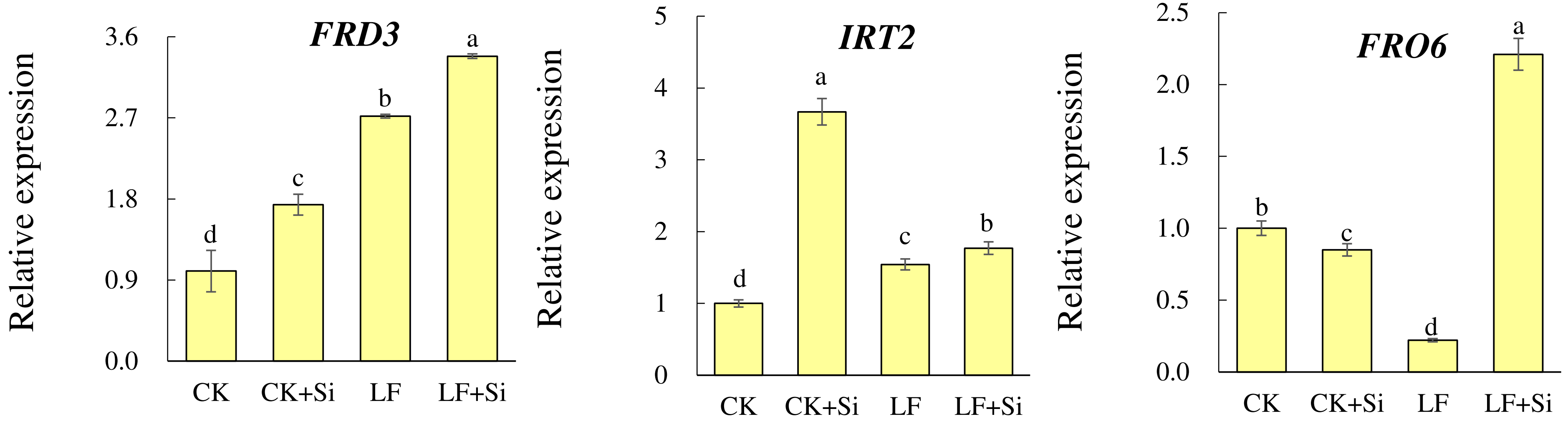

Supplementary Figure 3 The relative expression of some differentially expressed genes related to iron uptake and transport quantified by qRT-PCR. Each data point represents the mean of three independent biological replicates (mean ± SD). Different letters above the bars indicate statistically significant differences (P<0.05). Control (CK): 100 µmol/L Fe+ 0 mmol/L Si, Control and Si application (CK+Si): 100 µmol/L Fe + 1.5 mmol/L Si, Fe deficiency treatment (LF): 1 µmol/L Fe + 0 mmol/L Si, and Fe deficiency and Si application (LF+Si): 1 µmol/L Fe + 1.5 mmol/L Si.
